# The secreted *Theileria annulata* Ta9 protein contributes to activation of the AP-1 transcription factor

**DOI:** 10.1371/journal.pone.0196875

**Published:** 2018-05-08

**Authors:** Ahmet Hakan Unlu, Shahin Tajeri, Huseyin Bilgin Bilgic, Hasan Eren, Tulin Karagenc, Gordon Langsley

**Affiliations:** 1 Vocational School of Gevas, Van Yuzuncu Yil University, Van, Turkey; 2 Laboratoire de Biologie Cellulaire Comparative des Apicomplexes, Faculté de Médecine, Université Paris Descartes - Sorbonne Paris Cité, Paris, France; 3 Inserm U1016, Cnrs UMR8104, Cochin Institute, Paris, France; 4 Department of Pathobiology, Faculty of Veterinary Medicine, Ferdowsi University of Mashhad, Mashhad, Iran; 5 Department of Parasitology, Faculty of Veterinary Medicine, Adnan Menderes University, Aydin, Turkey; Centre National de la Recherche Scientifique, FRANCE

## Abstract

*Theileria annulata* is an obligate intracellular protozoan parasite of the phylum *Apicomplexa*. *Theileria* sporozoites invade bovine leukocytes and develop into a multinucleate syncytial macroschizont that causes uncontrolled proliferation and dissemination of infected and transformed leukocytes. Activator protein 1 (AP-1) is a transcription factor driving expression of genes involved in proliferation and dissemination and is therefore a key player in *Theileria*-induced leukocytes transformation. Ta9 possesses a signal peptide allowing it to be secreted into the infected leukocyte cytosol and be presented to CD8 T cells in the context of MHC class I. First, we confirmed that Ta9 is secreted into the infected leukocyte cytosol, and then we generated truncated versions of GFP-tagged Ta9 and tested their ability to activate AP-1 in non-infected HEK293T human kidney embryo cells. The ability to activate AP-1-driven transcription was found to reside in the C-terminal 100 amino acids of Ta9 distant to the N-terminally located epitopes recognised by CD8+ T cells. Secreted Ta9 has therefore, not only the ability to stimulate CD8+ T cells, but also the potential to activate AP-1-driven transcription and contribute to *T*. *annulata*-induced leukocyte transformation.

## Introduction

The protozoan parasite *T*. *annulata* is the causative agent of a tick-borne disease of cattle called tropical theileriosis that causes morbidity and loss of productivity in indigenous cattle, and a severe and often lethal disease in exotic and cross breed cattle in a wide geographic distribution ranging from the Mediterranean littoral regions of Europe and Africa to the Near and Middle East to India and China in Asia [1,2]. *T*. *annulata* is transmitted by ticks of the genus *Hyalomma* and during feeding on cattle infected ticks inject sporozoites that infect B cells and macrophages rather than T cells [3,4]. A unique aspect of *T*. *annulata* and *T*.*parva* infection is the ability of the macroschizont stage to induce host cell transformation without involving the integration of parasite DNA into the host genome [3]. *Theileria* is the only eukaryote known to transform another eukaryote [5] and *Theileria*-induced transformation of bovine leukocytes is reversible by killing the parasite with drug buparvaquone (Bw720c) [6].

There are many host cell factors that play a role in *Theileria*-induced transformation of bovine leukocytes such as activation of the transcription factors Myc (c-Myc) and Nuclear Factor-kappa B (NF-кB) that play an important role in inhibiting apoptotic death of infected cells [7,8]. AP-1 is another transcription factor that transcribes genes involved in a wide range of cellular processes such as differentiation, proliferation and dissemination that contribute to cellular transformation [9–11]. It has been previously shown that AP-1 activation in *Theileria*-transformed leukocytes is dependent on permanent c-Jun N terminal kinase (JNK) activity and upregulation of Jun and Fos family proteins [12]. There are many, often interacting, signalling pathways associated with *Theileria*-induced leukocyte transformation, but the precise initiating transformation event is unknown [13–15].

Bioinformatic analyses of the *T*. *annulata* genome revealed that TA15705 is member of a paralogous gene family made up of five members clustered on chromosome 2 [16]. Ta9 encoded by TA15705 is strongly expressed at the macroschizont stage and has both T- and B-cell epitopes that likely play roles in humoral and cytotoxic immune responses to the parasite [17,18]. Its DNA sequence (http://www.genedb.org/featureSeq/TA15705) predicts that TA15705 encodes a signal peptide consistent with Ta9-derived peptides being presented in the context of MHC class I to CD8+ lymphocytes [18]. Given that Ta9 appears to be secreted directly into the infected leukocyte cytosol (there’s no parasitophorous vacuole in *Theileria*-infected leukocytes) it could also play a role in *Theileria*-induced manipulation of host cell signalling. Consistent with this notion, microarray analyses performed on different developmental stages of *T*. *annulata* showed that of the different members of the paralogous family only Ta9 expression increased during macroschizont development and became reduced as the transforming macroschizont develops into merozoites that invade red blood cells [19].

As a secreted parasite factor Ta9 has the potential to play a role in host leukocyte transformation and so, we first used affinity purified anti-Ta9 antibodies to confirm the presence of Ta9 in the infected leukocyte cytosol and then expressed GFP-tagged truncated versions of Ta9 in non-infected HEK293T human kidney embryo cells to identify the region of Ta9 capable of activating the AP-1 transcription factor. The ability to activate AP-1-driven transcription was found to reside in the C-terminal 100 amino acids of Ta9.

## Materials and methods

### Cell culture

BL3 is a bovine B-lymphosarcoma cell line and TBL3 is an *in vitro* infection of Hissar stock of *T*. *annulata* were obtained from naturally infected cow and have been described [20]. These cell lines and the *T*. *annulata* infected cell line (Pendik) were cultured as described previously [21]. Human embryonic kidney (HEK293T) (Sigma, Germany) adherent cells used for transfections were cultured in a 10ml Dulbecco’s modified Eagle medium (DMEM, Invitrogen, France) containing 7.5% foetal calf serum (FCS), 100 u/ml Penicillin/Streptomycin and maintained at 37°C with 5% CO2 in air.

### PCR, restriction digestion and ligation

Ta9 (for DNA sequence see TA15705) and truncated sub-clones were generated in order to test their ability to activate AP-1-driven luciferase. Firstly, the original pcDNA3-Ta9 was isolated from a *T*. *annulata*-specific DNA library [16]. Truncated versions of Ta9 were designed based on the CD8+ T cell epitope regions [18]. PCR fragments were amplified using the following primers carrying restriction sites:

Ta9-1-335 aminoacids (aa)

5'-CCTACATAGCCCGGGATGAATCTCC-3' sense (*SmaI*)

5'-CTTCTCTAATCGATTTTCTAATCC-3' antisense (*ClaI*)

Ta9-44-335 aa

5'-CAAAGGAGTCCCGGGATGTTTGAGG-3' sense (*SmaI*)

5'-CTTCTCTAATCGATTTTCTAATCC-3' antisense (*ClaI*)

Ta9-1-101 aa

5'-CCTACATAGCCCGGGATGAATCTCC-3' sense (*SmaI*)

5'-GGACCTCCATCGATGCACCTCATGCCTCCTGATCG-3' antisense (*ClaI*)

Ta9-235-335 aa

5'-CCGGCCCGGGAAATTGGGA CCAGTAGACGC sense (*SmaI*)

5'-CCGGATCGATCTAATCCTTTTCTTCCCATGGTT antisense (*ClaI*)

Ta9 constructs were amplified with following PCR reactions; 10X buffer for KOD hot start DNA polymerase (Merck, USA), 25 mM MgSO_4_, dNTPs (10 mM each), PCR grade water, sense primer (10 μM), anti-sense primer (10 μM), Kod hot start DNA polymerase (1u/μl) and pcDNA3-Ta9 template. PCR set up performed with the following settings: Ta9-1-335 aa and Ta9-44-335 aa: 95°C for 5 min; followed by 5 cycles of 95°C for 20 s, 40°C for 10 s, and 70°C for 35 s; followed by 15 cycles of 95°C for 20 s, 45°C for 10 s, and 70°C for 35 s with a final extension period of 3 min at 68°C. Ta9-1-101 aa and Ta9-235-335 aa; 95°C for 5 min; followed by 5 cycles of 95°C for 20 s, 45°C for 10 s, and 68°C for 10 s; followed by 15 cycles of 95°C for 20 s, 55°C for 10 s, and 68°C for 10 s with a final extension period of 3 min at 68°C. The DNA was run in an agarose gel (1%) and visualised by addition of 10μl/ml of ethidium bromide. The SmartLadder (Eurogentec, Belgium) was used as a molecular weight marker. DNA fragments were purified with SV Gel and PCR Clean-Up System (Promega, USA) following the manufacturer’s protocol.

pLHC_M3_-EGFP plasmids (Invitrogen, France) and Ta9 PCR fragments were digested with *SmaI* at 25°C for 2 h (BioLabs, UK) and *ClaI* at 37°C for 2 h (BioLabs, UK) and run on an agarose gel (1%). The truncated fragments were purified with SV Gel and PCR Clean-Up System (Promega, USA) and eluted with 50μl nuclease free water. The DNA concentration was determined using NanoDrop (Thermo Scientific, USA). For the ligation reaction inserts and vector were ligated with T4 ligase (BioLabs, UK) and then transferred to competent DH5α *E*. *coli* (Invitrogen, France). Some of the colonies were selected for Miniprep (Promega, USA) and restriction digest following the manufacturer’s instructions.

### Control plasmids, reporter constructs and expression vectors

pLHC_M3_-EGFP vector used as a negative control. AP-1 luciferase reporter (3X-TRE-luc) promoter region consensus sequence has been described previously [22]. pUHD16-1 plasmids (lacZ under CMV promoter) were transfected together with control vector and expression vectors to measure β-Galactosidase activity that determines transfection efficiency. pLHC_M3_-EGFP-Ta9-1-335, pLHC_M3_-EGFP-Ta9-1-101, pLHC_M3_-EGFP-Ta9-44-335 and pLHC_M3_-EGFP-Ta9-235-335 vectors were respectively transfected together with 3X-TRE-Luc and pUHD16-1 plasmid to measure relative AP-1 activity.

### Transfection and measurement of AP-1 luc activity

HEK (Human Embryo Kidney) 293T (ATCC: CRL-11268) cells at 80–90% confluence were transfected with equal amounts (250 μg) of 3X-TRE luciferase reporter and β-galactosidase plasmids and 500 ng of other plasmids (totally 1 μg per condition) using X-tremeGENE 9 DNA Transfection Reagent (Roche, Germany) in 6-well cell culture plates. After 24h, the cells were lysed and Firefly luciferase and β-galactosidase activity were measured by the Dual-Light^®^ chemiluminescent reporter gene assay system (Applied Biosystems, USA) in a microplate luminometer (Berthold technologies, Centro LB960). AP-1 levels were normalised to β-galactosidase and reported. Two independent transfections were performed in triplicate. Remaining lysates were stored at -20 °C for western blot analysis.

### Antibody purification

Antibody purification was performed to confirm the subcellular location of Ta9. For this purpose full-length GFP-Ta9-1-335 was transfected HEK293T cells and extracts (500μg) deposited on 10% polyacrylamide gel and SDS polyacrylamide electrophoresis performed at 100 volt for 2 h. Electrophoretic transfer to a nitrocellulose membrane was performed at 4°C, 100 volt for1 h. Proteins were detected by Ponceau staining and the GFP-Ta9-1-335 protein bound to the membrane was cut out and the membrane blocked with PBS/Tween 0.1%, milk 5%. The membrane was incubated with 2 ml of *T*. *annulata* hyper-immune serum and washed 4-times with PBS/Tween 0.1%. Hyper-immune serum had previously been produced and described [23]. The membrane was cut into small pieces, incubated with 0.5 ml 0.2 M HCl glycine (pH 2.15) for 15 min and neutralized with 0.2 ml of 1M K_2_HPO_4_ (verified at pH7). Slide-A-Lyser Dialysis Cassette (Thermo Scientific, USA) was used for buffer exchange and this step was performed overnight in 1X PBS at 4°C. Concentration of purified Ta9 antibodies (250 μg/ml) were measured using a NanoDrop spectrophotometer (Thermo Scientific, USA). The specific Ta9 affinity purified antibodies were labelled with Alexa Fluor 594 Microscale Protein Labelling Kit A30008 (Invitrogen, France) for molecular imaging.

### Buparvaquone treatment

TBL3 and BL3 cells, at 2x10^5^ cells/ml were treated with 50 ng/ml theileriacidal drug buparvaquone (Bw720c) (stock 2 mg/ml in ethanol) for 48 h. Control samples treated with an equal volume of ethanol absolute were prepared. BL3 cells were used as a negative control and Ta9-GFP transfected HEK293T cells were used as a positive control. Protein expression level was compared with tubulin.

### Western blotting

The level of protein expression was calculated with regard to β-Galactosidase activity. Cell extracts (20μl) were mixed with 5μl of Laemmli dye solution (BioRad, USA) supplemented to contain 10% (vol/vol) mercaptoethanol and heated in a water bath at 95°C for 5 min. The mixture was deposited on 12% polyacrylamide gel and SDS polyacrylamide electrophoresis was performed at 100 volt for 2 h. A molecular weight marker (All Blue Standards, BioRad) was run concurrently as a size marker. Electrophoretic transfer was performed at 4°C, 100 volt for 1 hour in a transfer buffer using transblot transfer system (BioRad, USA). Antibodies used were:

Primary antibody: αGFP mouse (Jackson ImmunoResearch, UK), 1/1000 diluted in PBS/Tween 0,1%, BSA 3%, NaN3 0,05%

Primary antibody: Monoclonal anti-β-Tubulin (Sigma), 1/1000 diluted in PBS/Tween 0,1%, BSA 3%, NaN3 0,05%

Secondary antibody: Anti-Mouse IgG, Label: Peroxidase, Host: Goat, (Sigma) 1/10.000 diluted in PBS/Tween 0,1%, milk 5%.

Primary antibody: *T*. *annulata* hyper-immune serum (day 42) 1/6000 diluted in PBS/Tween 0,1%, fish gelatin 0,1%, NaN3 0,05%.

Primary antibody: Purified Ab of anti-Ta9, 1/250 diluted in PBS/Tween 0,1%, fish gelatin 0,1%, NaN3 0,05%.

Secondary antibody: Peroxidase-conjugated AffiniPure Goat Anti-Bovine IgG (H+L) (Jackson ImmunoResearch Lab) 1/200.000 diluted in PBS/Tween 0,1%, fish gelatin 0,5%.

### Immunofluorescence analyses

Glass coverslips were coated with an excess of 0.01% poly-L-lysine for at least 10 min. Cells remaining on coverslips were fixed by a 15 min incubation with 3% paraformaldehyde in PBS containing 2% sucrose. Coverslips were incubated 5 min in 0.2%PBS+TritonX-100 to permeabilize cells. For blocking, coverslips were incubated 15–20 min in 0.2% PBS+Triton X-100 with 5% FCS. Primary antibody was purified anti-Ta9 Ab (1μg/ml) and secondary antibody was FITC anti-bovine (Sigma, Germany) 1/500 diluted in 0.1% PBT with 5% FCS. Coverslips were incubated with 50μl of DAPI (Sigma, Germany) at 1/1000 in PBS with Ca^++^ and Mg^++^ for 5 min. Mowiol (Sigma, Germany) was used to mount each coverslip. Cells were examined by immunofluorescence microscopy (Leica DMI6000) and processed by using the Metamorph programme (Molecular Devices, USA).

## Results

### Ta9 is secreted into the infected leukocyte cytosol

**Fig 1:**
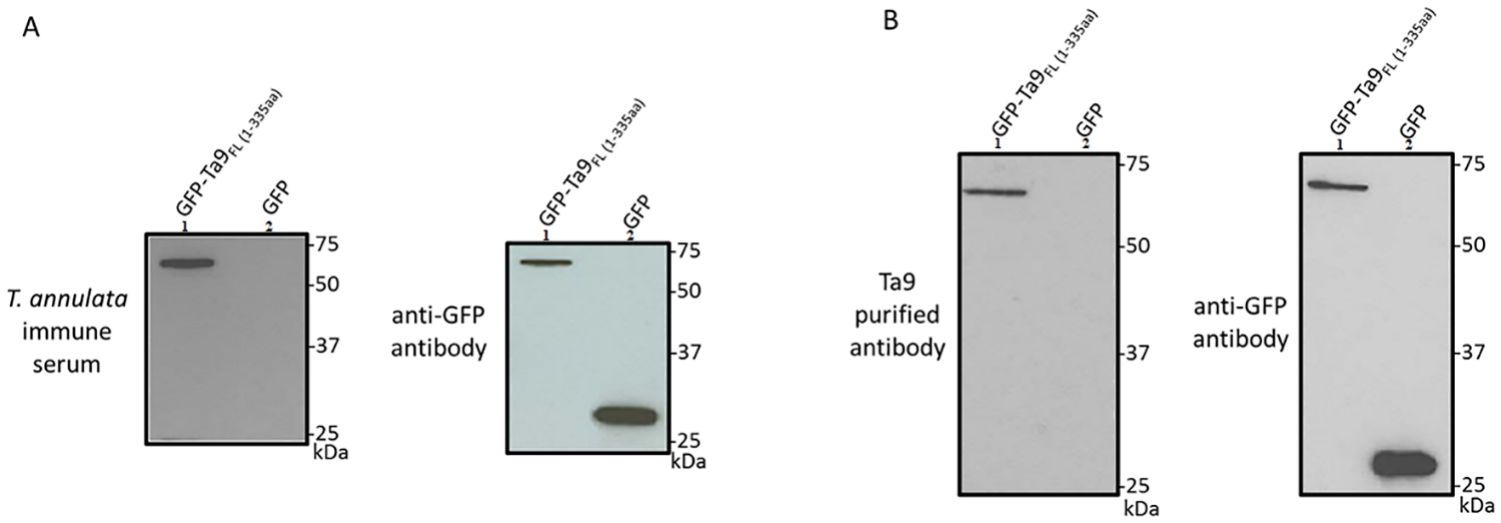
Western blot of GFP-Ta9 fusion protein detected with immune sera (A) and Ta9-specific affinity purified antibody (B). A: Lane 1; GFP-Ta9-1-335, 2; GFP-only. B: Lane 1; GFP-Ta9-1-335, 2; GFP-only. M: All Blue Standards (BioRad). Control anti-GFP antibody.

To confirm that the *T*. *annulata* hyper-immune serum contained specific Ta9 antibodies full-length GFP-Ta9-1-335 and a GFP-only plasmid were transfected into HEK293T cells and analysed by Western blot (Fig 1A). An anti-GFP antibody was used to control for GFP-Ta9 expression. Only GFP-Ta9-1-335 reacted with the hyper-immune serum and the fusion protein was used to affinity purify specific anti-Ta9 antibodies (Fig 1B) that were used in indirect immunofluorescence assays (IFA) to confirm the reported presence of Ta9 in the infected leukocyte cytosol [17,18].

**Fig 2:**
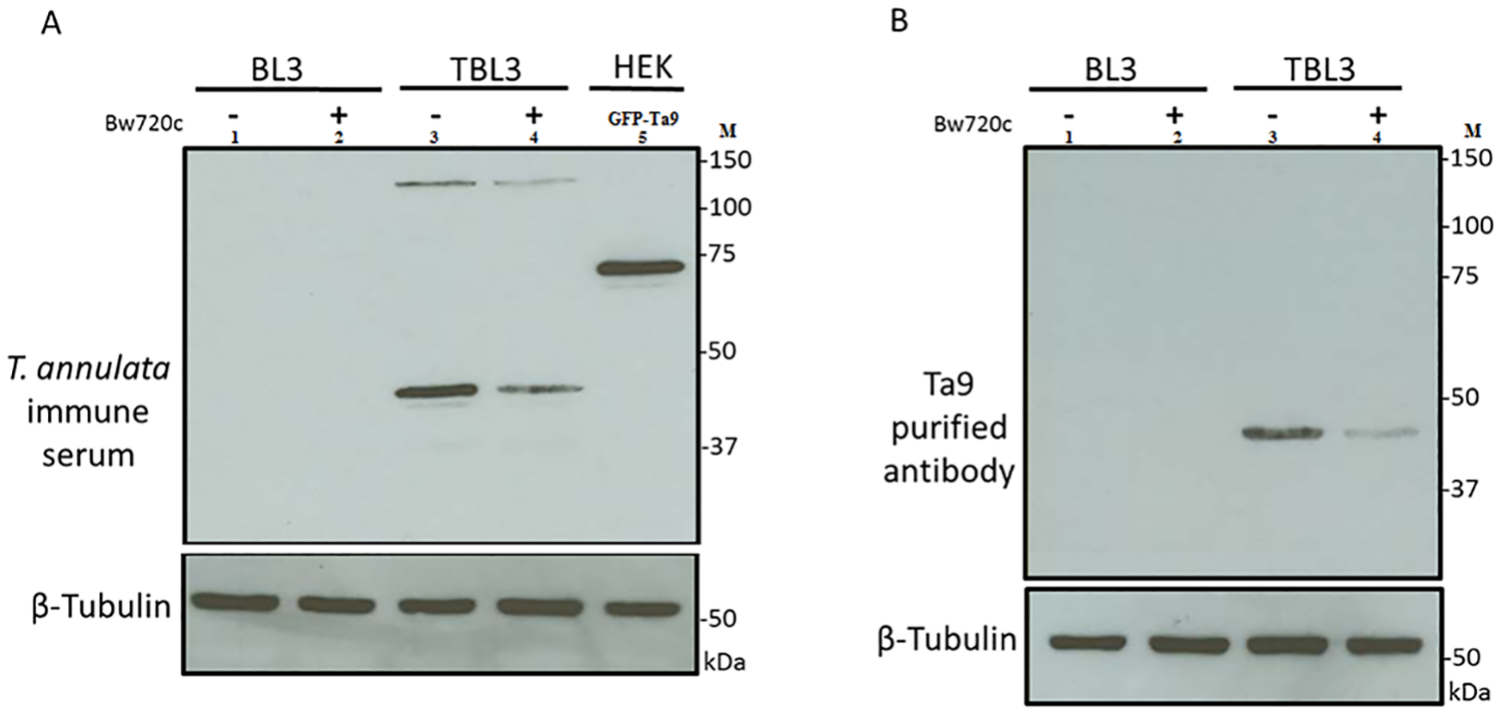
The level of Ta9 protein following Bw720c treatment. A: Hyper-immune sera; Lane 1: BL3 cells non-treated with Bw720c, 2: BL3 cells treated with Bw720c, 3: TBL3 cells non-treated with Bw720c, 4: TBL3 cells treated with Bw720c, 5: GFP-Ta9-1-335 aa. B: Ta9 specific affinity purified ab; Lane 1: BL3 cells non-treated with Bw720c, 2: BL3 cells treated with Bw720c, 3: TBL3 cells non-treated with Bw720c, 4: TBL3 cells treated with Bw720c. M: All Blue Standards (BioRad). Control anti-β-Tubulin.

To test the specificity of anti-Ta9 antibodies, TBL3 cells were treated 48 h with Bw720c and non-treated with Bw720c as a control. The level of Ta9 protein analysed by Western blot using Ta9 affinity purified antibodies and hyper-immune serum (Fig 2). When compared with non-treated TBL3 cells, Ta9 expression was decreased in drug-treated TBL3 cells. This is consistent with the amount of Ta9 being dependent on the live parasites.

**Fig 3:**
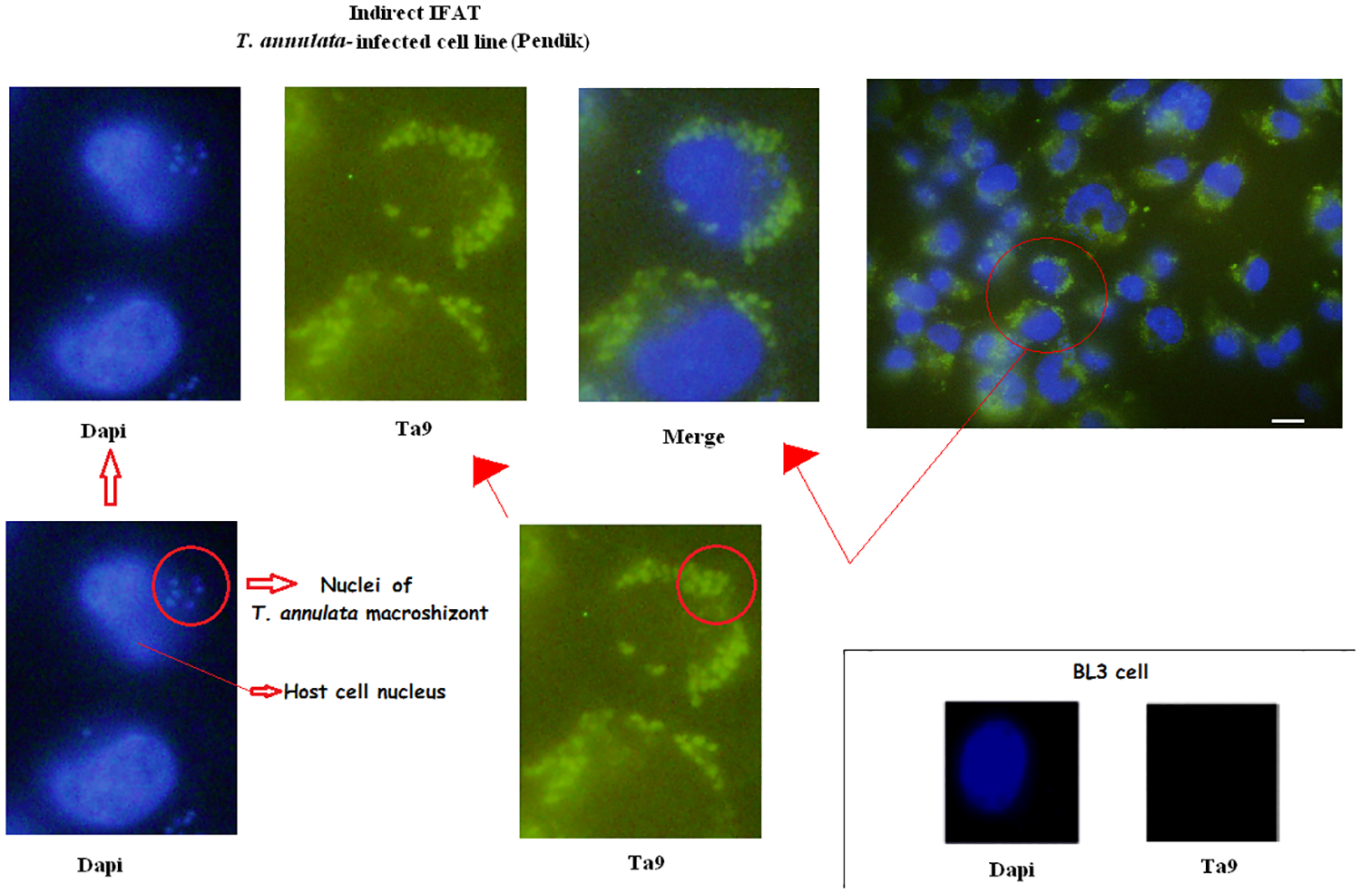
Subcellular localization of Ta9. Nucleus; DAPI (excitation 325 nm, emission 460 nm, exposure time 50 msec), Ta9 (excitation 590 nm, emission 617 nm, exposure time 500 msec). Scale bar 10 μm.

*T*. *annulata*-infected cell line (Pendik) was examined using the affinity purified Ta9-specific antibodies to confirm the presence of Ta9 in the infected leukocyte cytosol. Non-infected BL3 B cells were used as a negative control. As expected, the affinity purified anti-Ta9 antibodies both decorated the parasite and decorated Ta9 in the cytosol of *T*. *annulata*-infected cell (Fig 3).

### GFP-Ta9 expression in HEK293T cells activates AP-1-driven luciferase

**Fig 4:**
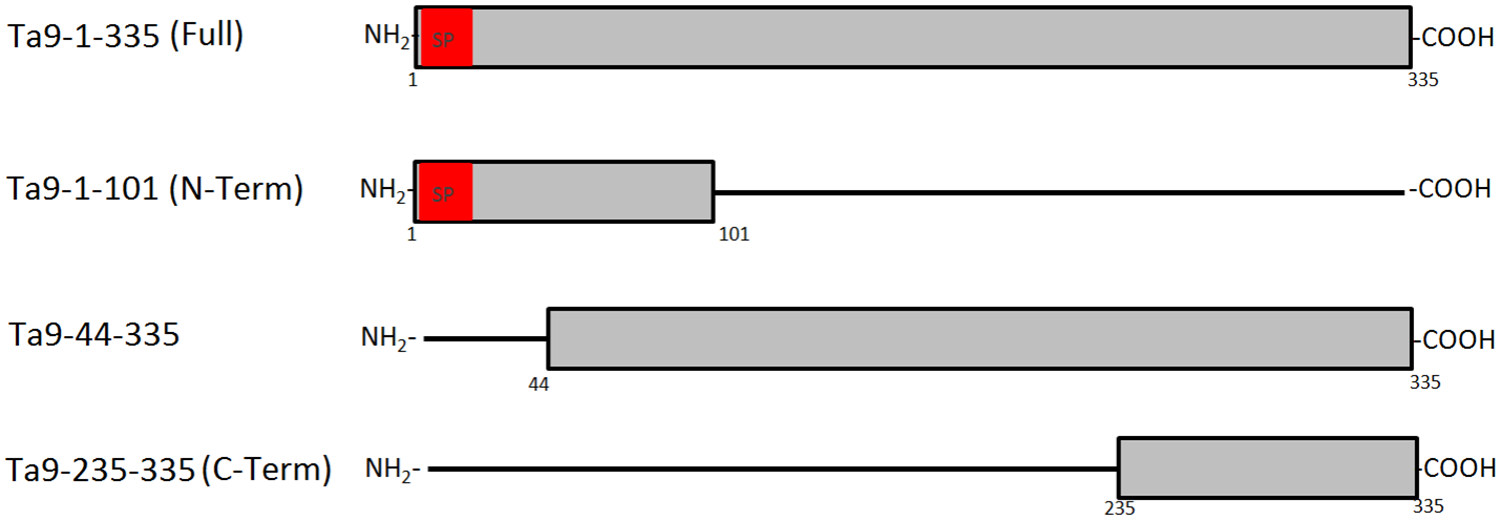
Ta9 constructs used for transfections.

**Fig 5:**
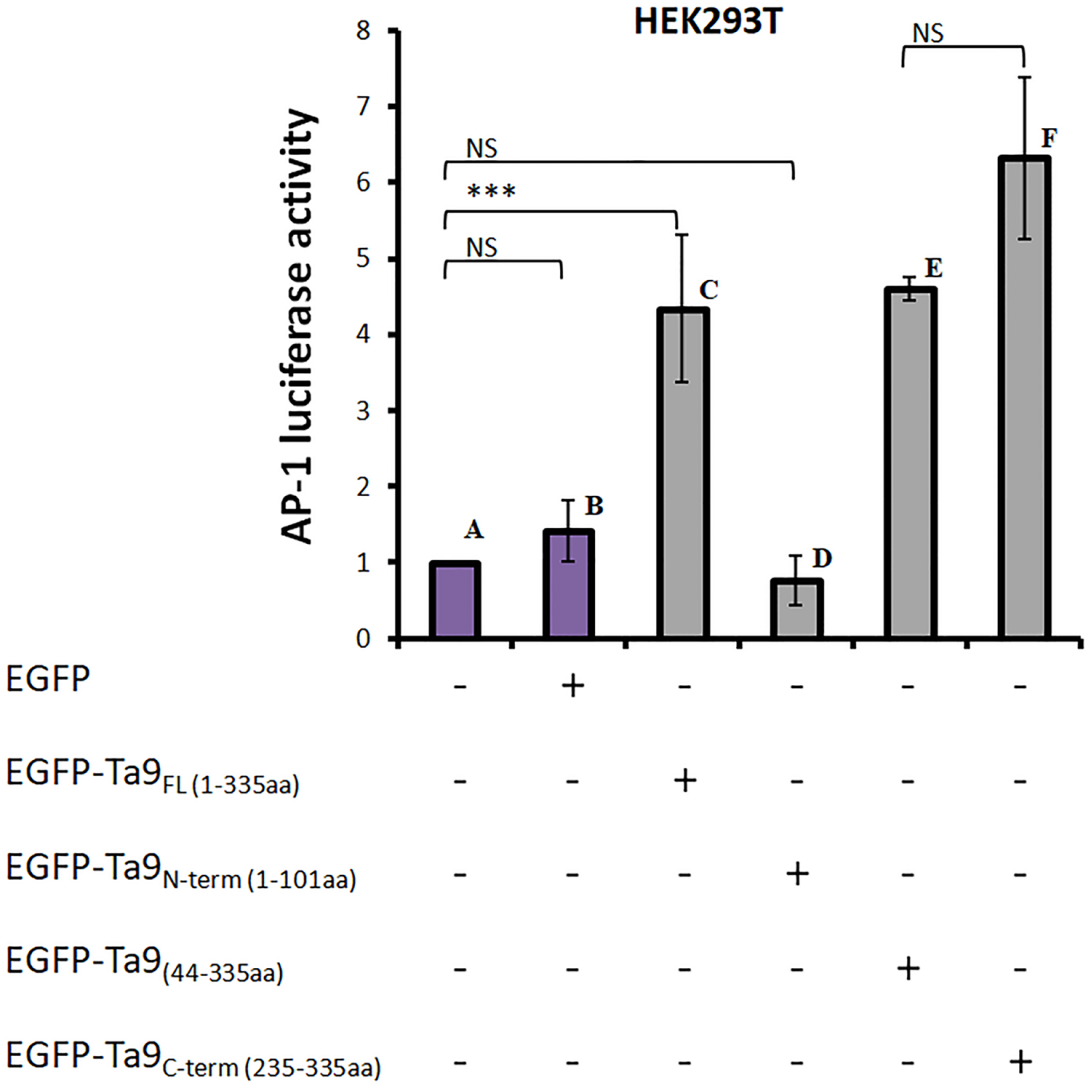
Ta9-induction of AP-1-driven luciferase activity in HEK293T cells. A: Only reporters, B: GFP with reporters, C: GFP-Ta9-1-335 aminoacids (aa) (full length) with reporters, D: GFP-Ta9-1-101 aa (N-terminally fused) with reporters, E: GFP-Ta9-44-335 aa with reporters, F: GFP-Ta9-235-335 aa (C-terminally fused) with reporters. *P* value was calculated by Student’s T-test. (n = 2, *p<0.05, ***p<0.001).

Having confirmed that Ta9 is secreted into the infected leukocyte cytosol, where in addition to be presented by the MHC [17,18] it also has the potential to subvert host cell signalling we tested its ability to activate AP-1-driven transcription. To exclude other secreted parasite factors contributing to activation of AP-1-driven transcription GFP-tagged Ta9 was transfected into non-infected HEK293T cells. To identify the region of Ta9 capable of activating AP-1 different length versions of Ta9 were N-terminally fused to GFP: full-length Ta9-1-335 amino acids, Ta9-44-335 amino acids and Ta9 C-terminal Ta9-235-335 amino acids (Fig 4). AP-1-driven luciferase and β-galactosidase reporter plasmids were co-transfected with each of four GFP-Ta9 expression constructs and AP-1-driven luciferase activity measured. The pLHC_M3_-EGFP vector used as negative control to give a measurement of the basal level of AP-1-driven transcriptional activity in HEK293T cells (Fig 5B). Expression of GFP-Ta9-235-335 fusion protein activated most AP-1-driven luciferase compared to GFP-Ta9-44-335 and GFP-Ta9-1-335 (Fig 5C, 5E and 5F). Expression of GFP-Ta9-1-101 gave background levels of AP-1-driven (Fig 5D). The ability to significantly induce AP-1-driven luciferase transcription appears to reside exclusively between amino acids 235 and 335 of Ta9.

## Discussion

The host cell transcription factor AP-1 has been demonstrated to play a role in the dissemination of transformed leukocytes infected with *Theileria* parasites, notably due to its capacity to drive transcription of the *mmp9* gene [12,24–26]. The observation that Ta9-derived peptides are recognised by CD8+ T cells already demonstrated that peptides of Ta9 containing CD8 epitopes are released into the infected leukocyte cytosol and presented by MHC-1 [18]. Using affinity purified anti-Ta9 antibodies we confirmed Ta9 is secreted into the infected leukocyte cytosol, and we now demonstrate that this location allows it to contribute to activating the AP-1 transcription factor.

Transfection of HEK293T cells with full-length GFP-Ta9-1-335 and truncated versions induced different levels of AP-1 activation. Full-length GFP-Ta9-1-335, GFP-Ta9-44-335, and the C-terminal fragment GFP-Ta9-235-335 were all capable of activating AP-1-driven luciferase (Fig 5). By contrast, the GFP-Ta9-1-101 fusion protein was unable to activate AP-1-driven luciferase and one concludes that the capacity of Ta9 to activate AP-1-driven luciferase resides between amino acids 235 and 335. Inspection of Ta9 for different linear motifs (elm.eu.org/) indicated the presence of two motifs relevant to the capacity of the C-terminal (235–335) fragment of Ta9 to activate AP-1-driven transcription. First, between amino acids 243 to 253 there’s a D-motif that’s present in MAP kinase binding proteins suggesting that Ta9 could interact via its D-motif with JNK kinase and in such a way activate JNK-signalling to induce AP-1. Second, between amino acids 320 to 327 there’s a monopartite variant of the classical nuclear localisation signal (NLS) suggesting that as Ta9 gets processed and loaded onto MHC class I [18] the C-terminal fragment could enter the host cell nucleus and interact either with activated JNK, or even directly with AP-1. Thus, we posit that the D-motif and/or the NLS present in the C-terminus of Ta9 facilitate Ta9 induction of AP-1-driven transcription, but specific antibodies to the C-terminal 100 amino acids will be necessary to observe whether this fragment enters the host cell nucleus.

*T*. *annulata* can target host cell proteins making up the heterodimeric AP-1 transcription factor. Secretion of a prolylisomerase, TaPIN1 (TA18945) provokes degradation of host ubiquitin ligase FBW7 leading to increased amounts of c-Jun (a subunit of AP-1 heterodimer) [27]. *Theileria*-infection also induces high levels of miR-155 that regulates a feedback loop leading to sustained c-Jun protein levels [28] and infection upregulates miR-126-5p levels leading to suppression of JIP-2 liberating JNK1 to translocate to the nucleus and phosphorylate c-Jun [29]. So, secretion of Ta9 and its ability to activate AP-1-driven transcription is yet another way that *Theileria* parasites manipulate host cell AP-1 activity underscoring the pivotal role that AP-1 plays in *Theileria*-induced leukocyte transformation.

The first 101 amino acids of Ta9 appear to lack B-cell epitopes, since the GFP-Ta9-1-101 fusion protein didn’t react with the hyper-immune serum. By contrast, this N-terminal region encodes CD8+ T cell epitopes demonstrated to be the target of CD8+ T cells in killing assays [18]. Clearly, Ta9 is a complex protein harbouring different regions, the N-terminal 101 amino acids posses cytotoxic CD8+ T cell epitopes, the central region variant B cell epitopes and the 100 amino acid C-terminal region capable of activating the AP-1 transcription factor. Future research will address how the 100 C-terminal amino acids of Ta9 activate AP-1-driven transcription.
